# The complex roles of neutrophils in APAP‐induced liver injury

**DOI:** 10.1111/cpr.13040

**Published:** 2021-05-04

**Authors:** Huiting Guo, Shiwei Chen, Mingjie Xie, Cheng Zhou, Min Zheng

**Affiliations:** ^1^ The State Key Laboratory for Diagnosis and Treatment of Infectious Diseases The First Affiliated Hospital Zhejiang University School of Medicine Hangzhou China; ^2^ Collaborative Innovation Center for Diagnosis and Treatment of Infectious Diseases Hangzhou China

## Abstract

Acetaminophen (APAP) is a widely applied drug for the alleviation of pain and fever, which is also a dose‐depedent toxin. APAP‐induced acute liver injury has become one of the primary causes of liver failure which is an increasingly serious threat to human health. Neutrophils are the major immune cells in human serving as the first barrier against the invasion of pathogen. It has been reported that neutrophils patriciate in the occurrence and development of APAP‐induced liver injury. However, evolving evidences suggest that neutrophils also contribute to tissue repair and actively orchestrate resolution of inflammation. Here, we addressed the complex roles in APAP‐induced liver injury on the basis of brief introduction of neutrophil's activation, recruitment and migration.

## Graphical abstract

A lot of factors deeply influence the neutrophil's activation, recruitment and migration in APAP‐induced liver injury. DAMPs like HMGB1 and ATP are released from damaged hepatocytes, recognized by RAGE, P2Y2 receptors, respectively, inducing the activation and recruitment of neutrophils. But the TLR9 expressed on neutrophils recognize mtDNA and then neutrophils could release miR‐223, which inhibits the neutrophil's activation and recruitment. As for other immune cells, they are divided into cells that promote and inhibit the activation and recruitment of neutrophils. Firstly, macrophages express OPN after APAP treatment and then attract neutrophils. Moreover, HMGB1 could recognize TLR4 receptor expressed on macrophages, release IL‐23 to induce the release of IL‐17A in γδ T cells and finally activate and recruit neutrophils. However, Ly6C^hi^ monocytes inhibit the activation and recruitment of neutrophils via CCR2 and M‐CSF pathways. Macrophages could also generate MerTK to inhibit neutrophils. Furthermore, TNF‐α/LPS MDSCs could express iNOS to decrease the intrahepatic neutrophils infiltration and induce the apoptosis of activated neutrophil. Activated and recruited neutrophils then migrate to the site of injury to alleviate the inflammation and induce tissue repair. GRPR antagonist could downregulate the expression of CD11b/CD66b via activating MAPKs pathways, which significantly influence the migration of neutrophils to the injury site.

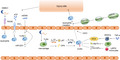

## 1 INTRODUCTION

Acetaminophen (APAP) is a kind of widely used drug for the treatment of pain and fever. It is reported that its overdose can cause serious liver failure.^1^ In Europe and the United States, APAP‐induced acute liver injury (ALI) has become one of the primary causes of liver failure and thus arises widespread concern and research in the world.^2^ Abundant evidences have suggested that both innate and adaptive immune cells, such as macrophages, neutrophils and T cells, played critical roles in APAP‐induced ALI.^3^, ^4^ It is now clear that neutrophils have important homeostatic functions in various organ systems including liver,^5^, ^6^ but what the role of neutrophils plays in APAP‐induced ALI still remains elusive.^7^, ^8^ Neutrophils, a type of polymorphonuclear leukocytes, are the first‐line guardians of the innate immune system. Normally, neutrophils are not activated and move slowly in peripheral blood circulation without direction. The half‐life of neutrophils is only 6‐7 hours.^9^ Once the pathogen invades or the endogenous stimulants releases, the pattern recognition receptors can recognize pathogen‐associated molecular patterns (PAMPs) and damage‐associated molecular patterns (DAMPs). Then, inflammatory reaction occurs and neutrophils are activated.^10^, ^11^ The activated neutrophils in peripheral circulation will tend to move towards the injury site. The migration of neutrophils involves rolling, activation and adhesion.^12^, ^13^ They then participate in the inflammatory process through phagocytosis, degranulation and extracellular traps (NETs).^14^ On the one hand, neutrophils are drawn from the blood to sterility inflammatory site, which contribute to wound healing. On the other hand, their prolonged half‐life, release of granule proteins such as myeloperoxidase (MPO), neutrophil serine proteases (NSPs), lipocalin 2 (LCN2), human neutrophil peptide (HNP), excessive infiltration and uncontrolled activation may lead to destruction of normal tissue structures and serious inflammations.^15^ Therefore, how neutrophils play in APAP‐induced ALI has aroused great controversy. In this review, we outline the neutrophil's complex roles in APAP‐induced ALI, focusing on its activation, recruitment and migration.

## 2 NEUTROPHIL'S ACTIVATION, RECRUITMENT AND MIGRATION IN APAP‐INDUCED LIVER INJURY

### 2.1 DAMPs

**FIGURE 1:**
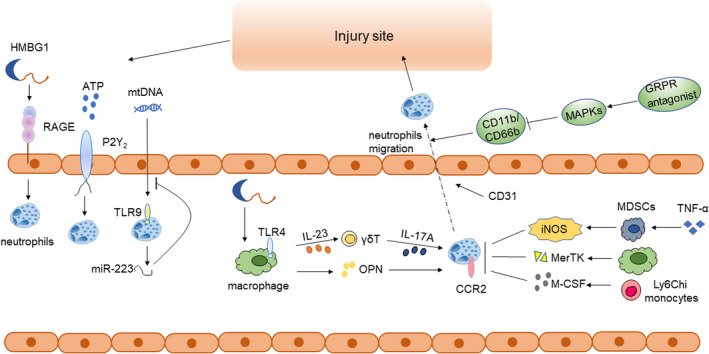
Schematic outline for the role of neutrophils in APAP‐induced ALI. DAMPs like HMGB1 and ATP are released from damaged hepatocytes, recognized by RAGE, P2Y2 receptors, respectively, inducing the activation and recruitment of neutrophils. But the TLR9 expressed on neutrophils recognize mtDNA and then neutrophils could release miR‐223, which inhibits the neutrophil's activation and recruitment. As for other immune cells, they are divided into cells that promote and inhibit the activation and recruitment of neutrophils. Firstly, macrophages express OPN after APAP treatment and then attract neutrophils. Moreover, HMGB1 could recognize TLR4 receptor expressed on macrophages, release IL‐23 to induce the release of IL‐17A in γδ T cells and finally activate and recruit neutrophils. However, Ly6C(hi) monocytes inhibit the activation and recruitment of neutrophils via CCR2 and M‐CSF pathways. Macrophages could also generate MerTK to inhibit neutrophils. Furthermore, TNF‐α/LPS MDSCs could express iNOS to decrease the intrahepatic neutrophils infiltration and induce the apoptosis of activated neutrophil. Activated and recruited neutrophils then migrate to the site of injury to alleviate the inflammation and induce tissue repair. GRPR antagonist could downregulate the expression of CD11b/CD66b via activating MAPKs pathways, which significantly influence the migration of neutrophils to the injury site

DAMPs are key signals which induce cell death in sterile inflammation including high‐mobility group box 1(HMGB1), ATP, ADP, IL‐1, IL‐33, heat shock proteins (HSPs) and so on. Epithelial HMGB1 triggered recruitment of neutrophils but not macrophages through its receptor RAGE, finally inducing necrosis after APAP treatment^16^ (Figure 1). In addition, HMGB1 could also mediate neutrophil infiltration via HMGB1‐TLR4‐IL‐23‐IL‐17A axis^17^ (Figure 1). After the administration of APAP, wild‐type C57BL/6 mice released abundant ATP and following increased the expression of P2Y2 receptors, which was required for the liver infiltration of neutrophils and subsequent liver injury^18^ (Figure 1). Mitochondrial DNA (mtDNA) released by damaged hepatocytes is another important stimulus which activates neutrophils via binding of Toll‐like receptor 9 (TLR9). However, mtDNA/TLR9 could also limit neutrophil overactivation through the negative feedback pathway of microRNA‐223 (miR‐223)^19^ (Figure 1).

### 2.2 Interplay with other immune cells

#### 2.2.1 Macrophages

Numerous pro‐inflammatory factors such as chemokines, IL‐1β and TNF‐α are released by activated liver macrophages, thus further aggravating inflammation and increasing neutrophils inflow.^20^ In addition, osteopontin (OPN), another pro‐inflammatory cytokine related to liver cell necrosis, was rarely expressed in Kupffer cells normally, but its expression increased significantly in hepatic macrophages at 6 hours after APAP administration, which attracted neutrophils to hepatic injury sites and caused massive liver necrosis (Figure 1).^21^ This result was further demonstrated in OPN−/− mice model, which exhibited less neutrophil infiltration and reduced expression of pro‐inflammatory cytokines in liver, such as IL‐1α and TNF‐α.^22^ Macrophages could also induce the generation of IL‐17–producing γδ T cells via the HMGB1‐TLR4‐IL‐23 pathway, enhancing the neutrophil infiltration and liver injury (Figure 1).^17^ However, some studies showed opposite results that macrophages could also inhibit the activation and recruitment of neutrophils. In APAP‐induced ALI mice model, injection of bone marrow‐derived macrophages significantly reduced HMGB1 translocation, infiltrating neutrophils and hepatocytes necrosis.^23^ It was also reported that increased MerTK+ macrophages could inhibit the continuous necrosis in APAP‐induced ALI by suppressing the activation of neutrophils and accelerating their clearance^24^ (Figure 1).

#### 2.2.2 Monocytes

Ly6C^hi^ monocytes controlled the activation and recruitment of neutrophils via CCR2(C‐C motif chemokine receptor 2) and M‐CSF pathways.^25^ In the livers of CCR2−/− mice, a significant increase in neutrophils could be observed at 24 hours following APAP administration. Moreover, the ablation of Ly6C^hi^ monocytes and their MoMF (monocyte‐derived macrophages) descendants leaded to a profound increase in neutrophil levels^26^ (Figure 1).

#### 2.2.3 Myeloid‐derived suppressor cells (MDSCs)

Myeloid‐derived suppressor cells (MDSCs), a heterogenous population of immune cells from myeloid lineage, can be enlarged during various pathological conditions, such as cancer and inflammatory diseases. Treated with various cytokines, bone marrow‐derived MDSCs can be differentiated to various types of cells with different functions. With APAP administration, tumour necrosis factor alpha/LPS‐primed MDSCs (TNF‐α/LPS MDSCs) could express iNOS to decrease the excessive intrahepatic neutrophil infiltration and induce the apoptosis of activated neutrophils, showing the strongest hepatic protective effect^27^ (Figure 1).

### 2.3 Chemokines and Cytokines

**FIGURE 2:**
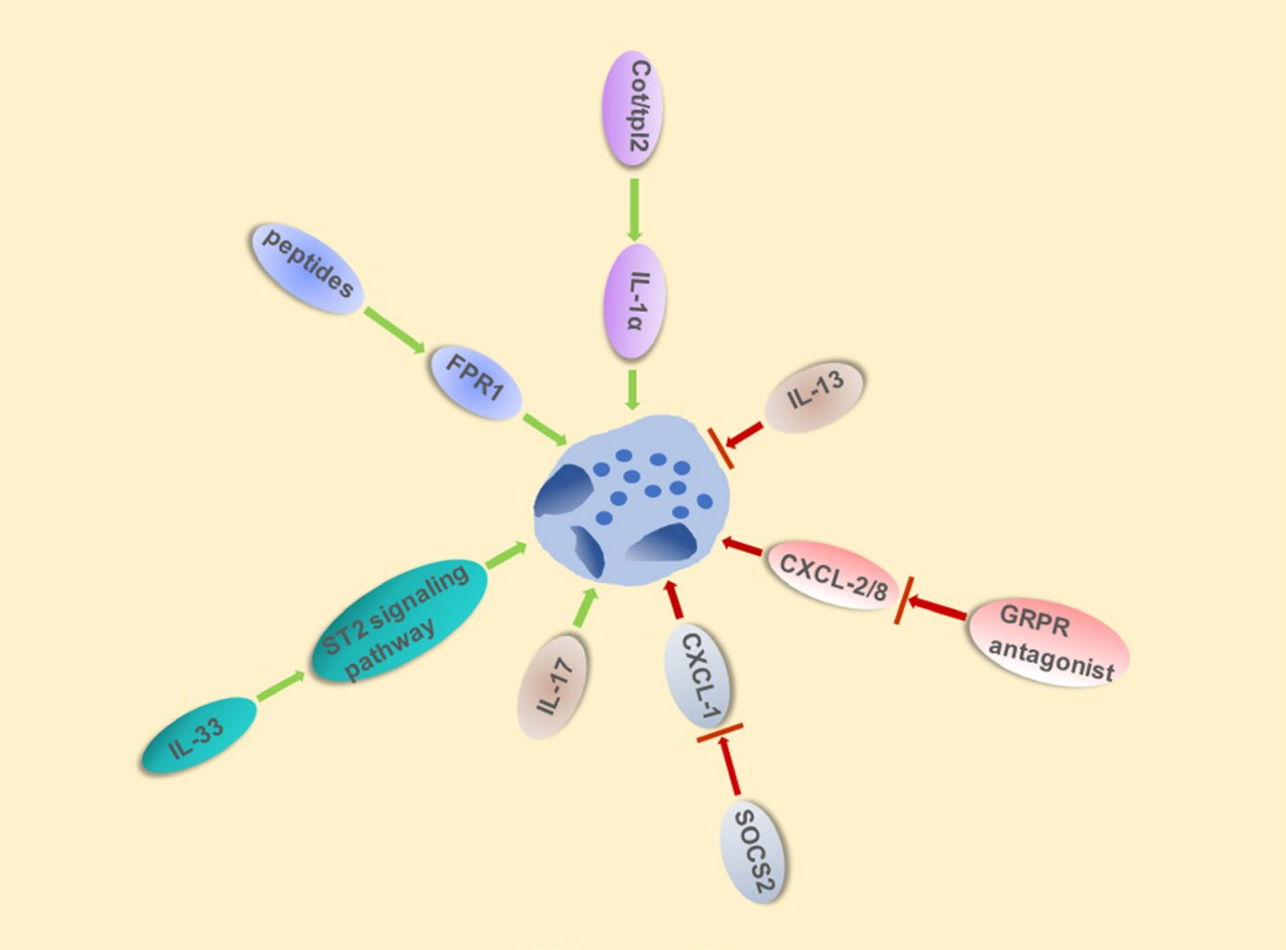
Chemokine and Cytokine influence activation, recruitment and migration of neutrophils in APAP‐induced ALI. Various chemokine and cytokine influence the activation and recruitment of neutrophils in APAP‐induced ALI. Mitochondria‐derived formyl peptides recruit neutrophils to sites of liver necrosis via FPR1. Similarly, IL‐33, IL‐17 and activated Cot/tpl2 induced neutrophils infiltration whereas IL‐13 significantly attenuates APAP‐induced liver injury via decreased neutrophils recruitment. SOCS2 and GRPR antagonist can also inhibited infiltration of neutrophils via downregulating CXCL‐1 and CXCL‐2/8, respectively

In addition to the interaction between immune cells contributes a lot in APAP‐induced hepatitis, some chemokines and cytokines also play an indispensable role. It was demonstrated that chemokines and mitochondria‐derived formyl peptides collaborated to recruit neutrophils to sites of liver necrosis via CXC chemokine receptor 2 (CXCR2) and formyl peptide receptor 1 (FPR1), respectively (Figure 2).^28^ As an important inhibitor of CXCL‐1, SOCS2 is able to control the activation and recruitment of neutrophils. In SOCS2−/− mice treated with APAP, the expression of the neutrophil‐active chemokine CXCL‐1 increased significantly, inducing more neutrophil recruitment and liver necrosis (Figure 2).^29^ Besides, in vivo, gastrin‐releasing peptide receptor (GRPR) antagonist inhibited both CXCL2‐induced neutrophil migration and activation through the downregulation of CD11b and CD62L. GRPR could also induce activation of MAPKs (p38 and ERK1/2) and downregulation expression of CD11b and CD66b, which significantly inhibited the adhesion and migration of neutrophils. In vitro, it decreased CXCL8‐driven neutrophil recruitment independently of CXCR2^30^ (Figure 2).

Some researchers believed that the cytokines released through inflammasome pathway also affected neutrophil's activation and recruitment. Imaeda et al mentioned that inhibition of the Nalp3 pathway with Aspirin and the knockout of Nalp3 in mice could decrease the infiltration of neutrophils and thus alleviate APAP‐induced liver injury.^31^, ^32^ However, C David Williams et al^33^ put forward the opposite results that after APAP treatment, mice deficient for each component of the Nalp3 inflammasome (caspase‐1, ASC and Nalp3) had similar neutrophil recruitment and liver injury when compared with that of WT mice, which appeared the limit effect of Nalp3 inflammasome. It is notable that these two studies all showed that plasma levels of IL‐1β protein are very minor even after APAP overdose. Moreover, Williams et al treated animals with pharmacological levels of murine recombinant IL‐1β and found that it had no effect on liver injury, which is consistent with IL‐1R−/− mice were not protected against APAP hepatotoxicity. These data together indicate that this minor increase in IL‐1β induced by APAP overdose did not aggravate liver injury. In addition, no increase expression of IL‐18 was observed in APAP‐induced liver injury.^33^ Furthermore, Williams et al repeat the aspirin pre‐treatment experiment and revealed that like many other interventions against neutrophils, Aspirin cannot protect liver from APAP‐induced injury through detecting HMGB1, K‐18 and other APAP metabolism bookmarks, which were absent in Imaeda study.^33^, ^34^, ^35^ As for the time point, Williams et al^33^ took their experiments at 24 hours instead of 12h in the Imaeda study, which should be taken into consideration when comparing their results. Above all, Nalp3 inflammasome signalling might not be a major pathway involved in APAP hepatotoxicity.

Other interleukins also play important roles in the activation and recruitment of neutrophils. The deficiency of IL‐13^36^ aggravated liver damage due to the neutrophils infiltration induced by injurious downstream events whereas absence of IL‐17^37^ significantly attenuated APAP‐induced liver injury via decreasing neutrophils recruitment (Figure 2). Acetaminophen overdose caused massive release of IL‐33 which can activate and recruit neutrophils, inducing liver injury by IL‐33/ST2 signalling pathway.^38^ In APAP‐induced ALI, activated Cot/tpl2 mediated the production of IL‐1α and IL‐1β, and the recruitment of neutrophils in response to DAMPs was largely dependent on IL‐1α^39^ (Figure 2).

CD31, also known as platelet endothelial cell adhesion molecule‐1 (PECAM‐1/ CD31), has pivotal function in the migration and clearance of neutrophils. The absence of CD31 could significantly alleviate APAP‐induced liver injury compared with WT mice.^40^

### 2.4 Time points of APAP overdose treatment

Neutrophils migrate into liver tissue in mice in the early stage of APAP‐induced liver injury occurrence(6,12 hours), and most were located at the healthy part of liver.^41^, ^42^ Then, the injury began to aggravate until it reached the peak at 24 hours with most of neutrophils migrated into the necrosis area. Subsequently, in the recovery phase of liver injury, neutrophils also began to decrease.

Some human's data showed that the phagocytic capacity and ROS production of neutrophils in patients with APAP‐induced liver injury were all increased during the recovery period (day 1‐day 4) which indicated that activation of neutrophils occurred after injury peaks. But there were also differences between human and mouse data. The difference was that the expression of CD11b is increased in peripheral blood neutrophils of mice, but not in humans, which may be caused by species variation and injury time.^42^

## 3 COMPLEX ROLES OF NEUTROPHILS

Abundant researches uncovered the pivotal roles of neutrophils in APAP‐induced liver injury, a few researchers believe that neutrophils contribute a lot in aggravating APAP‐induced liver damage. It was reported that neutrophils depletion by RB6‐8C5 (an anti‐Gr‐1 antibody) moderated APAP‐induced ALI, which indicated neutrophils induced liver injury. Yuko Ishida et al also confirmed this result. In vivo, they found improved survival rate in neutropenic WT mice and CXC chemokine receptor 2 (CXCR2)‐deficient mice when compared with WT mice under the same dose of APAP treatment. In vitro, isolated human neutrophils were toxic to HepG2 cells when cocultured via direct contact with HepG2 cells and the CXCR2‐FPR1‐signalling pathway.^28^, ^43^ Researchers further confirmed that neutrophil activation occurred secondary to the initial liver injury induced by APAP. They found that resolvins which can prevent and reduce the infiltration of neutrophils in the inflammation site could extend the therapeutic window after APAP administration. Collectively, these results indicate that neutrophils mediate (at least partially) the hepatotoxic effects of oral acetaminophen.^44^, ^45^

However, some researchers hold different views. Lawson et al reported that neutrophils participate in necrotic debris removal instead of affecting the pathogenesis of APAP‐induced ALI directly.^46^ It was further proved by Williams, C. D et al^47^ that upregulation of CD11b or reactive oxygen was not observed on neutrophils isolated from the liver after APAP administration. Moreover, hepatic neutrophils accumulation and activation caused by high pharmacological doses of IL‐1beta do not worsen APAP‐induced ALI.^48^ Other researchers showed that β2 integrins(CD11/CD18) is essential for neutrophils in the transmigration and adherence steps in mice liver. But treatment with anti‐CD18 monoclonal antibody had no protective effects on liver damage after APAP administration during the 24h time period, suggesting that neutrophils do not contribute to the initiation or progression of APAP‐induced ALI.^41^ Clapperton et al^49^ further showed a neutrophil defect in ALF due to overdose of paracetamol, which is complement‐dependent, but has nothing to do with serum complement, and may be related to complement receptors.

In addition, with the growing evidence that neutrophils actively control the regression of inflammation and contribute to tissue repair, the perception of the indiscriminate killers of neutrophils seems to be changed. Some researchers have also proposed the repair role of neutrophils in APAP‐induce ALI. Researchers found evidence that APAP treatment could lead to the delay of previously activated neutrophil apoptosis, which is beneficial to the repair of injured liver tissue. Freitas, M. et al^50^ also confirmed this view for the longevity of neutrophils was found to be prolonged after APAP administration, which helps tissues heal and resolve inflammation. Mechanically, neutrophils play a pivotal role in liver repair by promoting the conversion of pro‐inflammatory Ly6C^hi^CX_3_CR1^lo^ monocyte/macrophage phenotype into pro‐degradable Ly6C^lo^CX_3_CR1^hi^ macrophages.^51^ Furthermore, reactive oxygen species (ROS), mainly expressed by neutrophils in APAP‐induced liver injury, are important mediators that trigger the repair phenotype transformation of macrophages to promote liver healing.^52^ Recent studies further revealed that blocking platelet CLEC‐2 signalling increased the production of tumour necrosis factor alpha (TNF‐α), which then further accelerated the recruitment of reparative hepatic neutrophils, thereby promoting liver recovery from acute APAP‐induced injuries.^53^ These studies all strongly confirmed the indispensable role of neutrophils in tissue repair and regeneration.

## 4 CONCLUSION

As outlined in this review, we review the neutrophil's activation, recruitment, migration and the complex roles in APAP‐induced liver injury. There are different views about the roles of neutrophils involved in APAP‐induce liver injury. Some researchers have shown that neutrophils aggravate APAP‐induced liver damage, while others demonstrated that neutrophils are not involved in this damage. Several studies revealed that neutrophils even have repair functions in this liver hepatitis model. These quite controversial opinions might be related to time point of experimental observation. The timing of APAP treatment should be taken into account because the pathophysiology of APAP‐induced liver injury consists of several different stages which suggests that we should not use a single time point as the experimental observation point. It is necessary to investigate injury events at several time points within 24 hours and evaluate regeneration within 96 hours.^54^ Besides, different susceptibility of various mouse strains to APAP hepatotoxicity has been confirmed which need to be taken into consideration.^55^ What's more, off‐target effects in drugs or biological reagents used in APAP relevant researches should not be ignored.^56^ Thus, in order to clarify this elusive question, further studies need to be more detailed concerning the impact on APAP metabolism and intracellular signalling events of different intervention strategies.^54^

But undeniably, the studies of neutrophils also cast light in the prevention and treatment of APAP‐induced ALI. Neutrophil elastase (NE) is a pro‐inflammatory protein that secreted by activated neutrophils. The combination treatment of NAC (N‐acetyl‐L‐cysteine) and sivelestat (the inhibitor of NE) significantly attenuated liver damage. This strategy is more effective than NAC monotherapy and might provide new options for the treatment of APAP‐ALI.^57^ Besides, A large number of studies have shown that some molecules and signal pathways were involved in reducing APAP‐induced liver damage by inhibiting the recruitment and infiltration of neutrophils, such as Berberine (BBR),^58^ Geniposide (GP),^59^ BLT1 signalling,^60^ Glycyrrhetinic acid (GA),^61^ Citral,^62^ lemongrass essential oil (LGO),^63^ SmKI‐1^64^ and β‐d‐glucan.^65^ Therefore, it has been proposed that neutrophils can be considered as a target for the treatment of APAP‐induced ALI. Through clarification on the relationship of neutrophils with APAP‐induced inflammation may provide new strategies for the prevention and treatment of APAP‐induced ALI.

## CONFLICTS OF INTEREST

The authors declare no financial conflicts of interests.

## AUTHOR CONTRIBUTIONS

Huiting Guo wrote the manuscript and prepared figures; Min Zheng, Cheng Zhou, Shiwei Chen and Mingjie Xie provided expert comments and edits. All authors reviewed the manuscript.

## Data Availability

The data that support the findings of this study are available from the corresponding author upon reasonable request.
